# Characterization of a novel and spontaneous mouse model of inflammatory arthritis

**DOI:** 10.1186/ar3399

**Published:** 2011-07-12

**Authors:** Iris A Adipue, Joel T Wilcox, Cody King, Carolyn AY Rice, Katherine M Shaum, Cory M Suard, Elri ten Brink, Stephen D Miller, Eileen J McMahon

**Affiliations:** 1 Department of Biology, Westmont College, 955 La Paz Road, Santa Barbara, CA 93108, USA; 2 Department of Microbiology-Immunology and the Interdepartmental Immunobiology Center, Northwestern University Medical School, 303 East Chicago Avenue, Chicago, IL 60611, USA

## Abstract

**Introduction:**

Mouse models of rheumatoid arthritis (RA) have proven critical for identifying genetic and cellular mechanisms of the disease. Upon discovering mice in our breeding colony that had spontaneously developed inflamed joints reminiscent of RA, we established the novel IIJ (inherited inflamed joints) strain. The purpose of this study was to characterize the histopathological, clinical, genetic and immunological properties of the disease.

**Methods:**

To begin the IIJ strain, an arthritic male mouse was crossed with SJL/J females. Inheritance of the phenotype was then tracked by intercrossing, backcrossing and outcrossing to other inbred strains. The histopathology of the joints and extraarticular organ systems was examined. Serum cytokines and immunoglobulins (Igs) were measured by ELISA and cytometric bead array. Transfer experiments tested whether disease could be mediated by serum alone. Finally, the cellular joint infiltrate and the composition of secondary lymphoid organs were examined by immunohistochemistry and flow cytometry.

**Results:**

After nine generations of intercrossing, the total incidence of arthritis was 33% (304 of 932 mice), with females being affected more than males (38% vs. 28%; *P *< 0.001). Swelling, most notably in the large distal joints, typically became evident at an early age (mean age of 52 days). In addition to the joint pathology, which included bone and cartilage erosion, synovial hyperproliferation and a robust cellular infiltration of mostly Gr-1^+ ^neutrophils, there was also evidence of systemic inflammation. IL-6 was elevated in the sera of recently arthritic mice, and extraarticular inflammation was observed histologically in multiple organs. Total serum Ig and IgG1 levels were significantly elevated in arthritic mice, and autoantibodies such as rheumatoid factor and Ig reactive to joint components (collagen type II and joint homogenate) were also detected. Nevertheless, serum failed to transfer disease. A high percentage of double-negative (CD4^-^CD8^-^) CD3^+ ^TCRα/β^+ ^T cells in the lymphoid organs of arthritic IIJ mice suggested significant disruption in the T-cell compartment.

**Conclusions:**

Overall, these data identify the IIJ strain as a new murine model of inflammatory, possibly autoimmune, arthritis. The IIJ strain is similar, both histologically and serologically, to RA and other murine models of autoimmune arthritis. It may prove particularly useful for understanding the female bias in autoimmune diseases.

## Introduction

Rheumatoid arthritis (RA) is a systemic and chronic disease. The most characteristic symptoms are severe synovial inflammation, cartilage and/or bone destruction and bony nodule formation in the diarthrodial joints, but extraarticular manifestations are also prevalent and include pericarditis and vasculitis as well as pulmonary complications such as pleuritis and pulmonary fibrosis [1-3]. The worldwide prevalence of RA among adults is estimated to be between 0.3% and 2%, with more women affected than men [4-6]. Although the prevalence of RA does increase with age, the majority of adults with RA are diagnosed between the ages of 30 and 50 years. A juvenile form of disease, with onset before 16 years of age, does exist, and while differences in classification and diagnosis make estimates difficult, the prevalence of RA among children likely ranges between 0.007% and 0.4% worldwide [7]. RA is a debilitating disease associated with increased mortality and a decrease in survival by 3 to 10 years [8]. It is also a major cause of inability to work, with a little over one-third of RA patients reporting an inability to work within five years of diagnosis [9].

Unlike many other forms of arthritis, RA is autoimmune in nature. Autoantibodies are found in the vast majority of patients. While B-cell autoantigens include collagen type II, citrullinated proteins and glucose-6-phosphate isomerase, the classic example is rheumatoid factor (RF), an autoantibody specific for the Fc portion of immunoglobulin G (IgG) [10]. RF is found in 70% to 80% of adult RA patients and has been used as a diagnostic indicator for the past five decades [11]. While B cells have been successful targets for therapy in at least a subset of patients [10,12,13], the focus has shifted more toward T cells as the primary lymphocyte driving disease [11,14-17]. Drugs that limit T-cell activation help to ameliorate disease, and many of the genes associated with an increased susceptibility to RA are involved in T-cell function [11,18]. Though RA was once described as "Th1-driven," evidence is mounting that Th17 cells are the primary T-helper cell subset promoting disease [16,17,19].

While lymphocytes account for the autoreactivity in RA, the inflammation itself results from a plethora of cytokines that recruit peripheral immune cells, promote synovial proliferation and induce osteoclast maturation to increase bone resorption [11]. Many proinflammatory cytokines, such as TNF-α and IL-6, have been the targets of therapeutic biologic drugs that successfully limit inflammation [20]. Secreted primarily by activated macrophages, TNF-α is believed to have a particularly central role, as it induces the release of many other cytokines and chemokines and activates many of the cell types present in the inflamed synovium, including polymorphonuclear cells, endothelial cells, chondrocytes and osteoclasts [21]. IL-6 is similarly known for its pleiotropic functions [22]. It is the primary cytokine responsible for the acute phase response observed in RA, contributing to systemic inflammation [21], and strongly influences lymphocyte maturation, promoting both plasma cell and Th17 differentiation [23,24].

Mouse models of RA have been critical for understanding the underlying genetic and cellular mechanisms of pathogenesis [25-27]. Both induced and spontaneous models have been developed. In the most common experimentally induced models, injection of adjuvant and various joint matrix components, such as collagen type II or proteoglycan aggrecan, causes autoimmune arthritis in susceptible inbred mouse strains [28,29]. Spontaneous models can be subdivided by their mode of origination: (1) development of autoimmune-prone strains by selective mixing of previously existing inbred strains (for example, the MRL/lpr strain [30,31]), (2) targeted gene manipulation (for example, T-cell receptor (TCR)-transgenic K/BxN model [32], TNF-α overexpression models [33], IL-1Ra-knockout [34] and gp130^Y759F^-induced mutant [35]) and (3) identification of spontaneous mutants from breeding colonies (for example, SKG mice with a point mutation in Zap-70 [36]). While these models replicate many aspects of human RA, none of them mimic the disease completely. In fact, it is unlikely that RA itself is a single disease, but instead may be subdivided into various subsets (for example, RF-seropositive and RF-seronegative) with distinct etiologies. Therefore, each mouse model is potentially important not only for reinforcing the existence of common or even universal mechanisms of disease but also for revealing unique aspects not yet reflected in the other models.

In this report, we describe a new mouse line that spontaneously develops chronic inflammatory arthritis most evident in the large distal joints. Called the IIJ (inherited inflamed joints) strain, it was derived from arthritic (AR) animals discovered in a breeding colony at Northwestern University. One male AR mouse of predominantly SJL/J background was used to begin the line, and, after 13 generations of inbreeding, the phenotype remains stable. RA incidence in the entire colony is approximately 33%, with females developing arthritis more often than males. Joint histology confirmed cartilage and bone erosion, synovial proliferation and robust leukocyte infiltration. In addition to the inheritance pattern and histopathology, we describe the clinical and immunological properties of disease.

## Materials and methods

### Mice

The IIJ strain was established from AR mice that appeared in the 5B6 transgenic mouse-breeding colony at Northwestern University. 5B6 mice were initially developed at Harvard University [37]. While generated on the FVB background, they were backcrossed for five generations to SJL/J before being shipped to Northwestern University, where they were then crossed with Thy1.1 congenic SJL/J mice (backcrossed more than 12 generations to SJL/J). A subset of mice with inflamed joints was discovered after several months. One AR male mouse that was negative for the 5B6 TCR transgene was crossed with SJL/J female mice to generate the F1 generation of the IIJ line. Since then, the line has been maintained with sibling-sibling mating. SJL/J mice (six to nine weeks old) were purchased from Harlan Laboratories (Indianapolis, IN, USA) or Taconic Farms (Hudson, NY, USA) and used either directly in experiments or as breeders. For inbred strain crosses, five- to six-week-old female BALB/c, C3H, DBA1 and FVB mice were purchased from Harlan Laboratories.

Mice were housed in a specific pathogen-free (SPF) facility at Northwestern University or in a HEPA-filtered SuperMouse 750 ventilated rack and caging system (Lab Products, Inc., Seaford, DE, USA) at Westmont College. Antibiotic-treated mice were fed a modified Laboratory Rodent Diet 5001 supplemented with 0.06% amoxicillin, 0.0138% metronidazole, 0.0037% bismuth and 0.0004% omeprazole (Newco Distributors, Rancho Cucamonga, CA, USA) for eight weeks. All protocols performed were approved by the Institutional Animal Care and Use Committee of Northwestern University and the Institutional Review Board of Westmont College.

### Evaluation of clinical disease

Clinical disease was evaluated using a subjective scoring method similar to previous published methods [34,38] with minor modifications. On a weekly basis, each paw was assigned a score from 0 to 4 (0 = no disease; 1 = minor, localized swelling; 2 = moderate swelling involving majority of paw; 3 = major swelling with paws two to three times normal size; and 4 = deformity in large or small joints of paw). Scores from one reading were added together for the cumulative clinical score. Mice were considered AR when they maintained a score ≥ 2 for one paw for two consecutive readings. Disease onset was considered to be the first day that swelling was evident (score ≥ 1). For a subset of animals, disease was also tracked weekly by measuring ankle thickness with calipers. For each mouse, values for all four paws were added together to calculate the cumulative ankle thickness. At the time of joint scoring, the presence of other clinical symptoms such as evidence of colitis was also noted.

### Histopathology and immunocytochemistry

Joints were prepared for H & E staining according to previously published work [39]. Briefly, mice were killed, and their paws were removed, fixed in 10% formalin, decalcified and submerged again in 10% formalin until processed for paraffin embedding. Tissue processing, cutting of paraffin sections and H & E staining were performed at AML Laboratories, Inc. (Rosedale, MD, USA). In addition, live mice (five AR and two NAR IIJ mice) were sent to the Comparative Pathology Laboratory at the University of California, Davis. An animal necropsy was performed, and numerous organs and tissues were harvested for analysis. Sections were examined and scored for inflammation in a blinded fashion by a trained veterinary histopathologist.

Frozen tissue sections were prepared and stained according to previously published methods [40]. Serial frozen sections were cut and stained using biotinylated primary antibodies or isotype controls (eBioscience, San Diego, CA, USA) and the Tyramide Signal Amplification Kit (PerkinElmer, Waltham, MA, USA). They were analyzed using a Leica DM5000 B fluorescence microscope (Leica Microsystems, Buffalo Grove, IL, USA and SPOT Advanced Software (SPOT Imaging Solutions, Sterling Heights, MI, USA).

### Serum isolation

Mice were given a lethal dose of Euthanasia 5 solution (Henry Schein, Inc., Port Washington, NY, USA), and blood was collected via cardiac puncture. Blood was allowed to clot for a minimum of seven minutes at room temperature. It was spun for 10 minutes at 10,000 rpm in a tabletop microcentrifuge. The serum was removed and immediately frozen at -80°C until used.

### Cytokine assays

The mouse inflammation cytometric bead array (CBA) assay was performed according to the manufacturer's instructions (BD Biosciences, San Jose, CA, USA). Serum was diluted 1:4 with assay diluent. The samples were analyzed immediately using a FACSAria III cell sorter and FACSDiva software (BD Biosciences). The standard curves were made and calculations were performed using the BD Biosciences CBA software. Serum IL-17A levels were measured by ELISA using the manufacturer's protocol (eBioscience). Serum was diluted 1:5.

### Cell isolation and flow cytometry

Cells from lymphoid organs (spleens and lymph nodes) were isolated as previously described [41]. The leg-draining lymph nodes used included the axillary, brachial, inguinal and popliteal. The joints were removed and placed into a Petri dish containing 3 mL of sterile DMEM. After mincing the joints with a sterile razor blade, 1 mg/mL type II collagenase (Worthington Biochemical Corp., Lakewood, NJ, USA) and 100 μg/mL DNAse (Sigma-Aldrich, St Louis, MO, USA) were added. After incubating the samples for one hour at 37°C, a rubber plunger was used to push the tissue through a sterile metal mesh in a Petri dish to generate a single-cell suspension. The mesh was washed twice with 5 mL of Hanks' buffered saline solution (HBSS). Samples were centrifuged for five minutes at 500 × *g *at 4°C, resuspended in 5 mL of HBSS and strained through a 70-μm cell strainer. Live cells were counted in a hemocytometer using trypan blue exclusion.

Staining for flow cytometry was performed in 96-well U-bottomed plates. Cells (0.5 to 1 × 10^6 ^cells/well) were resuspended in 2% FCS in PBS, and a 1:100 dilution of anti-mouse CD16/32 (eBioscience) was added. Cells were incubated on ice for 15 minutes before antibody mixtures were added for staining. All antibodies were purchased from eBioscience (anti-mouse CD3ε-PE-Cy7, CD3ε-APC, CD4-FITC, CD8-APC, CD11b-PE, CD45-FITC, B220-APC, Gr-1-Pacific Blue, TCRβ-PE, Vβ6-FITC and Foxp3-APC), and their concentrations were individually optimized in comparison to an isotype control. The anti-mouse DX5-PE antibody was purchased from BD Biosciences. Cells were then incubated for 30 minutes on ice and washed three times with PBS. Cells were resuspended in PBS and analyzed immediately or fixed in 1% paraformaldehyde, stored overnight at 4°C and analyzed the next day. Intracellular Foxp3 staining was done using the Foxp3 Staining Buffer Set (eBioscience) according to the manufacturer's instructions. Data were collected on a FACSAria flow cytometer and analyzed using FACSDiva software (BD Biosciences) or FlowJo software (TreeStar, Inc., Ashland, OR, USA).

### Serum immunoglobulin ELISA

Total serum Ig, IgG1 and IgG2a levels were determined by performing a sandwich ELISA based on the manufacturer's published protocols (eBioscience). Plates were coated with 2 μg/mL of polyclonal goat anti-mouse Ig (553998; BD Biosciences) diluted in PBS. Serum was diluted in 5% FCS/PBS at 1:200,000 for total Ig, 1:50,000 for IgG1 and 1:40,000 for IgG2b. The standards were serial dilutions of mouse reference serum (Bethyl Laboratories, Montgomery, TX, USA). Antibodies were detected using 0.5 μg/mL biotinylated detection antibodies (total Ig, 553999; IgG1, 553441; and IgG2a, 553388; BD Biosciences), a 1:1,000 dilution of avidin-horseradish peroxidase (eBioscience) and Super AquaBlue ELISA Substrate (eBioscience). Concentrations were determined using the standard curve and dilution factor. Duplicates were averaged.

Total RF (that is, autoantibodies to IgG, IgM or IgA isotypes specific for the Fc portion of IgG) was measured using a commercial kit following the manufacturer's instructions (Alpha Diagnostic International Inc., San Antonio, TX, USA). Serum was diluted 1:250. Each sample was run in duplicate and averaged. To normalize between runs, the results are reported as percentages of the manufacturer-provided positive controls [(Average Abs_sample_/Average Abs_+control_) × 100].

Other antigen-specific total Ig ELISAs were performed by first coating high-binding 96-well plates with either 20 μg/mL collagen type II (Sigma) or 15 to 20 μg/mL joint homogenate (kindly provided by Dr Alison Finnegan). After the wells were washed, serum was diluted 1:5 in PBS and added to wells in duplicate. Reactivity was detected using the biotinylated anti-mouse Ig, avidin-horseradish peroxidase and Super AquaBlue ELISA Substrate mentioned above. Duplicates were averaged, and the results are reported as relative absorbance.

### Serum transfers

Serum was pooled and pushed through a 0.2-μm syringe filter to remove any contaminating cells. For each SJL/J recipient, 300 μL of serum were injected intraperitoneally. Mice were tracked for disease for a minimum of seven weeks. Mice were scored every two days for the first four weeks and then scored twice weekly thereafter.

### Statistics

Comparisons of disease incidence between groups were analyzed using Fisher's exact test. Means in the Ig ELISAs, cytokine assays and T-cell subset analyses were compared using an unpaired *t*-test. The mean percentages of various cell types in joint or lymphoid organ samples were compared by two-way analysis of variance.

## Results

### Occurrence of arthritis in a mouse-breeding colony and the establishment of the IIJ strain

**Figure 1:**
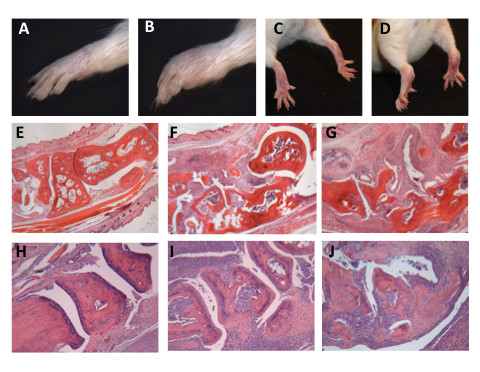
**Robust inflammation in the fore- and hindpaws of arthritic IIJ mice**. **(A) **and **(B) **Forepaws and **(C) **and **(D) **hindpaws of **(B) **and **(D) **arthritic (AR) and **(A) **and **(C) **nonarthritic (NAR) IIJ littermates. Mice were > 16 weeks old. **(E) **through **(J) **H & E-stained sectionsof formalin-fixed and decalcified paws. **(E) **through **(G) **Hindpaws (total magnification, ×40). **(E) **NAR littermate. **(F) **46-day-old AR mouse 7 days after onset. **(G) **141-day-old AR mouse 103 days after onset. **(H) **through **(J) **Forepaws (total magnification, ×100). **(H) **NAR littermate. **(I) **58-day-old AR mouse 26 days after onset. **(J) **261-day-old AR mouse 233 days after onset. Histological studies are representative of samples taken from nine AR and seven NAR littermates.

5B6 mice transgenic for a TCR specific for a myelin peptide (proteolipid proteins 139 to 151) were brought to Northwestern University and crossed with Thy1.1.SJL/J congenics. After several generations, it was observed that a small portion of the mice used as breeders had noticeable swelling in their large distal joints, a hallmark of disease in murine models of arthritis. The inflammation was observed in both the forelimbs (Figures 1A and 1B) and the hindlimbs (Figures 1C and 1D). This phenotype had not been reported in the 5B6 breeding colony at Harvard University, the institution at which the transgenic mice were generated, or in any report describing the use of Thy1.1.SJL congenics. Nevertheless, the arthritis phenotype appeared to be heritable, as a portion of the progeny from the breeders also developed inflamed joints. Arthritis occurred independently of the expression of the transgenic 5B6 TCR, since the peripheral blood T cells in some of the AR mice lacked high expression of Vβ6, a variable region used in the transgenic TCR (data not shown). An AR male mouse that lacked expression of the transgenic TCR but expressed Thy1.1 was crossed with SJL female mice to establish a new line, the IIJ strain. The strain has been maintained by brother-sister mating, and all mice used in this study were derived from this cross. IIJ mice from the F10 and F11 generations were also screened for Vβ6 expression in their peripheral blood T cells and similarly lacked the high expression characteristic of 5B6 mice (data not shown). Furthermore, mice from the IIJ line never showed signs of experimental autoimmune encephalomyelitis (EAE), the disease that spontaneously occurs in the 5B6 strain [37], making presence of the TCR transgenes highly unlikely.

H & E staining of sagittal sections of paws revealed histopathology consistent with inflammatory arthritis (Figures 1E, 1F, 1G, 1H, 1I, 1J). Both cartilage and bone erosion was observed, along with notable inflammatory infiltration, synovial proliferation and pannus formation. A veterinary histopathologist examined the sections from five AR IIJ mice (ranging from 98 to 277 days old) and two NAR littermates in a blinded fashion. While no inflammation was reported in the NAR mice, it was evident in multiple joints of the AR mice, including all the joints in the paws (phalangeal, metacarpal/tarsal and carpal/tarsal), the stifle (or "knee") joint and, occasionally, the vertebral joints. The cellular infiltrate was largely described as neutrophilic and histocytic and was severe enough to cause abscess formation in some of the most severely AR mice. Myeloid hyperplasia was also observed in the bone marrow.

### Non-Mendelian inheritance of the arthritis phenotype and influence of genetic background

**Figure 2:**
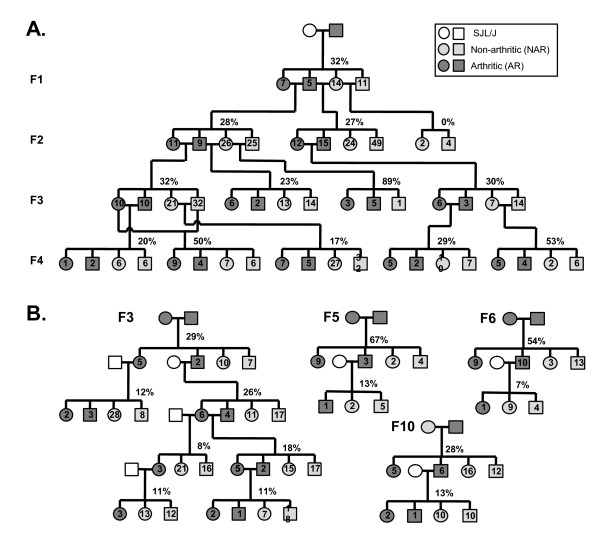
**Complex pattern of inheritance of arthritis phenotype in the IIJ strain**. Pedigrees representing the inheritance of the arthritis phenotype in multiple generations of IIJ mice after intercrossing or backcrossing. The arthritis incidence in a particular phenotypic cross in each generation is shown as a percentage above the progeny. The number inside each symbol represents the number of progeny of that sex and phenotype. For conciseness, the results of a particular phenotypic cross were combined within a line for each generation. **(A) **Pedigree of the first four generations of the IIJ strain. One of the nontransgenic arthritic males from the 5B6 colony was crossed with SJL/J females to establish the IIJ strain. The strain was then maintained by sibling-sibling mating. **(B) **AR IIJ mice from various generations (noted as F3, F5 and F6 next to cross) were backcrossed to SJL/J, and the progeny were tracked for disease.

A complex pattern of inheritance of the arthritis phenotype was observed in the IIJ strain (Figure 2A). The initial cross between an AR male mouse and SJL/J female mice produced F1 progeny with a disease incidence of 32% (12 of 37), suggesting a possibly dominant phenotype. However, crosses involving two AR F1 mice or an AR F1 male and a NAR F1 female produced progeny with a similar incidence (just under 30%), a general pattern that was maintained in later generations. Only one litter was produced from a cross between two NAR F1 mice, and none of the progeny developed arthritis (zero of six). However, in later generations (F2 to F4), NAR × NAR crosses consistently produced AR progeny (incidence ranging from 17% to 89%, depending on breeder).

After nine generations, the AR phenotype was stable, but the incidence remained at 33% overall (304 of 932) (Table 1). Furthermore, the incidence was close to one-third, regardless of the phenotype of either parent, although it was slightly higher when the dam was AR (198 (35%) of 562) than when the dam was NAR (106 (29%) of 370), regardless of the sire's phenotype (*P *< 0.05). The phenotype did appear to be sex-influenced, as female mice had a higher incidence than male mice (38% vs. 28%; *P *< 0.001). This pattern was observed regardless of the phenotype of the parents, although it was statistically significant only in the AR × AR group (*P *< 0.05). Taken together, these data indicate that the arthritis phenotype is not inherited in a simple Mendelian fashion and that females are more susceptible to it than males.

**Table 1:** Combined incidence of arthritis in first nine generations of IIJ strain^a^

		Progeny	
		
Phenotype of parents^b^	Total	Female	Male
Male × Female, *n *(%)			
AR × AR	159 of 463 (34%)	84 of 207 (41%)	75 of 256 (29%)
AR × NAR	77 of 273 (28%)	41 of 120 (34%)	36 of 153 (24%)
NAR × AR	39 of 99 (39%)	22 of 53 (42%)	17 of 46 (37%)
NAR × NAR	29 of 97 (30%)	15 of 44 (34%)	14 of 53 (26%)
Overall, *n *(%)	304 of 932 (33%)	162 of 424 (38%)	142 of 508 (28%)

^a^Progeny were considered arthritic when they maintained a score of 2 (see Materials and methods for details) on the same paw for two consecutive readings. Results were combined based on phenotype of parents regardless of generation. ^b^Arthritic (AR) or nonarthritic (NAR); all were intercrosses between littermates.

Multiple attempts were made to backcross AR IIJ mice further to SJL/J to determine whether the arthritis incidence could be increased if the contaminating FVB genes were removed (Figure 2B). Male and female AR mice from the F4 or later generations were separately crossed with SJL/J mice, and the progeny were tracked for disease. After consistently backcrossing or backcrossing followed by intercrossing, the incidence only decreased. It also decreased regardless of whether female or male AR IIJ mice were used for backcrossing. Therefore, FVB alleles likely contributed to the AR phenotype.

To further test the influence of genetic background on arthritis incidence, AR IIJ mice were also crossed with FVB and three other pro-AR inbred strains: BALB/c and CH3, two strains susceptible to proteoglycan-induced arthritis [29], and DBA1, a strain susceptible to collagen-induced arthritis [28]. AR male IIJ mice were crossed with two inbred strain females (two breeders per strain), and their progeny were tracked for disease. Arthritis incidence was minimal in all four strains, with only one IIJ × FVB progeny and one IIJ × C3H progeny developing disease (Table 2). When F1 siblings were intercrossed to generate a second generation, arthritis incidence again was low, never exceeding 8%. Therefore, since backcrossing to SJL or to other inbred strains only decreased arthritis incidence, the line was maintained by sibling-sibling intercrossing.

**Table 2:** Low incidence of disease when arthritic IIJ mice were crossed with four other inbred strains^a^

Cross^b^	Cage	F1	F2
AR × BALB/c	1	0 of 13	2 of 25
	2	0 of 18	0 of 19
AR × FVB	1	0 of 13	0 of 23
	2	1 of 22	2 of 38
AR × DBA1	1	0 of 20	0 of 21
	2	0 of 13	1 of 20
AR × C3H	1	1 of 16	0 of 24
	2	0 of 15	0 of 25

^a^Mice were followed for an average of 120 postnatal days and a minimum of 90 postnatal days, except in rare cases (< 2.5%) when males were killed earlier because of fighting with cagemates. ^b^Arthritic (AR) males from the IIJ strain were crossed with females from inbred strains. All arthritic F1 mice were used to generate the F2 generation.

### Progression of clinical disease varied between arthritic IIJ mice

**Figure 3:**
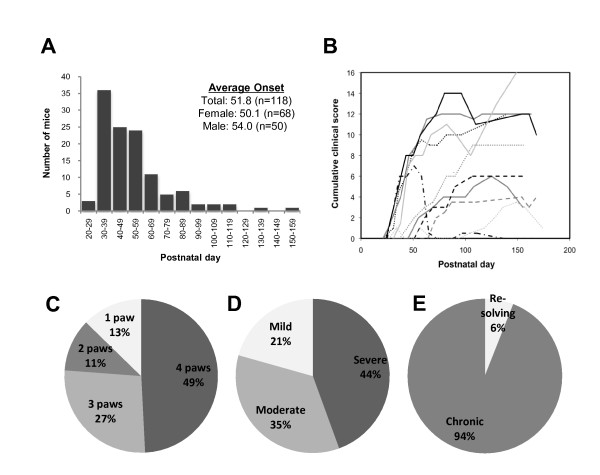
**Onset, progression, severity and persistence of clinical disease**. AR mice (*N *= 63; females = 34, males = 29) in the first four generations were followed for a minimum of 126 postnatal days (pnd) (average = 157 pnd) with a minimum of eight weekly readings (average = 12). The mice were scored on a weekly basis for the severity of clinical disease (see Materials and methods for details). **(A) **Histogram showing pnd at onset of clinical disease. While mice were classified as AR when they maintained a score of 2 for two consecutive readings, onset was designated as the first score ≥ 1. **(B) **Progression of cumulative clinical scores over time for selected AR mice. **(C) **The maximum numbers of paws affected are shown. Affected paws had a score ≥ 1. **(D) **The maximum severity of disease based on peak of cumulative clinical score is shown (mild = 2 to 5, moderate = 6 to 10 and severe = 11 to 16). **(E) **Chronic vs. resolving disease is shown. An AR mouse was classified as having chronic disease when it had a cumulative clinical score ≥ 2 for every weekly reading after onset. Resolving disease was deemed to have occurred when the cumulative joint score dropped below 2 and disease never recurred.

AR mice in the first four generations of the IIJ line were tracked for progression of clinical disease for a minimum of four months. Mice were scored weekly for disease on each paw, and the scores for each paw were added together to calculate the cumulative clinical score. A subset was also measured with calipers for ankle thickness (data not shown), which showed that AR mice had an increased cumulative ankle thickness compared to NAR mice and corroborated the subjective scoring method. On average, mice developed arthritis at approximately 7.5 weeks of age (51.8 postnatal days (pnd)), though onset ranged from 21 pnd (that is, arthritis at weaning) to > 150 pnd (Figure 3A). The age at onset was not significantly different between male and female mice. Once becoming classified as AR, most mice maintained that status throughout their lifetime. Clinical disease resolved in only 6% of the animals (Figure 3E). Clinical progression, total number of paws affected and peak severity did vary widely between mice (Figures 3B, 3C, 3D). While almost one-half of the AR mice developed inflammation on all four paws, one-fourth of them developed inflammation in only one or two paws, showing an occasionally asymmetric progression. Similarly, the peak severity of disease ranged from severe (44% with peak cumulative joint scores between 11 and 16) to moderate (35% with peak scores between 6 and 10), to mild (21% with peak scores between 2 and 5). Examples of disease progression for individual mice are shown in Figure 3B. Overall, clinical disease showed significant variability between mice.

### Typical signatures of local and systemic inflammation in arthritic IIJ mice

**Figure 4:**
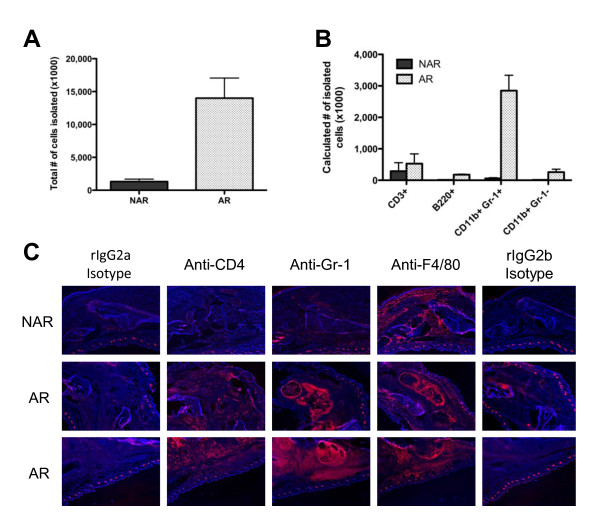
**Flow cytometric and immunohistochemical analyses of cell types present in the inflamed joints of IIJ mice**. **(A) **and **(B) **Cells were isolated from the inflamed paws of AR mice and the corresponding noninflamed paws of NAR littermates. Cells were stained with antibody mixtures and analyzed by five-color flow cytometry (*n *= 5 to 6 mice/group). **(A) **Average number of cells isolated. Error bars represent standard error of the mean (SEM). **(B) **Average number of cells of specific immune lineages (CD3^+ ^= T cell, B220^+ ^= B cell, CD11b^+^Gr-1^+ ^= neutrophil, CD11b^+^Gr-1^- ^= macrophage). Error bars represent SEM. After gating on live, CD45^+ ^cells, the percentages of each cell type were determined. This value was then multiplied by the total cells isolated from that animal to determine the final number of each cell type. **(C) **Immunohistochemical staining of serial sections of hindpaws from AR and NAR mice. Frozen sections were stained with biotinylated primary or isotype control antibody and detected using the Tyramide Signal Amplification Kit from PerkinElmer (staining shown in pink). The rat immunoglobulin 2a (Ig2a) isotype is the control for the anti-CD4 stain. The rat Ig2b isotype is the control for the anti-Gr-1 and anti-F4/80 stains. The mounting media contained 4',6-diamidino-2-phenylindole (staining shown in blue) for nuclear stain. AR mice were between 80 and 95 days old.

While H & E staining clearly showed robust cellular infiltration into the joints of AR IIJ mice, flow cytometry and immunohistochemistry were used to identify the types of immune cells present. Cells were isolated from the joints of AR and NAR littermates using collagenase digestion. Not surprisingly, the average number of cells isolated from joints of AR IIJ mice was over 10 times greater than the average number isolated from NAR littermates (14 million vs. 1.3 million) (Figure 4A). Flow cytometry revealed that the vast majority of immune cells (CD45^+^) were neutrophils (CD11b^+^Gr-1^+^), though T cells (CD3^+^), B cells (B220^+^) and macrophages (CD11b^+^Gr-1^-^) were also present at low levels (Figure 4B). Immunohistochemistry confirmed that Gr-1^+ ^neutrophils were the primary cell type in the inflamed joints, where they could be seen concentrating in abscesses (Figure 4C). Some CD4^+ ^T cells could also be seen in the inflamed joints, and while F4/80^+ ^myeloid cells could be seen throughout the healthy joints, these were presumably tissue-resident cells, and their numbers increased and changed in distribution in the inflamed joints.

**Figure 5:**
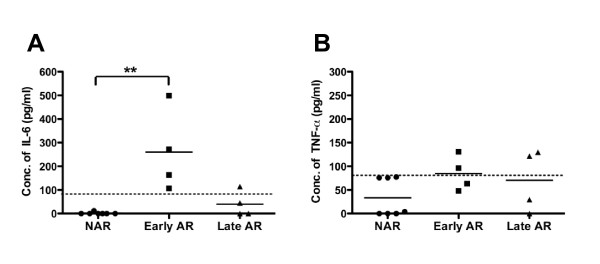
**Cytokine levels in serum of nonarthritic IIJ mice and arthritic IIJ mice at early and late stages of disease**. Serum was isolated from AR and NAR littermates, and the levels of six cytokines were measured by cytometric bead array. Only **(A) **IL-6 and **(B) **TNF-α were detected. IL-12p70, IL-10, monocyte chemotactic protein 1 and IFNγ were measured but not detected. Early AR mice were < 2.5 months old and had had arthritis for < 5 weeks. Late AR mice were > 4 months old and had had arthritis for > 11 weeks. Dashed horizontal line represents the minimum level of detection. Solid horizontal line represents the average per group. **P < 0.01. Filled circles, squares, and triangles represent data points from NAR. Early AR, and Late AR mice, respectively. Serum IL-17A was measured by standard ELISA but was not detected (data not shown).

To determine whether typical proinflammatory cytokines were elevated in the AR IIJ mice, serum was collected from AR mice at early and late stages of disease and from NAR littermates, and a CBA assay was performed to determine the level of six inflammatory cytokines. Though IL-12p70, monocyte chemotactic protein 1, IFN-γ and IL-10 were undetectable in all animals, IL-6 was elevated in all of the AR mice at early stages of disease (Figure 5A). TNF-α was only detected in four of eight AR, but none of the mice had levels twice the minimal level of detection of the assay (Figure 5B). Serum IL-17A was measured by ELISA but was also undetectable. Taken together, the significant infiltration of immune cells into the joints and the presence of IL-6 in the sera of AR mice are indicative of the robust inflammation occurring in the AR mice, both locally in the joint and systemically.

### Though serum Ig and autoantibodies were elevated in arthritic IIJ mice, serum failed to transfer disease

**Figure 6:**
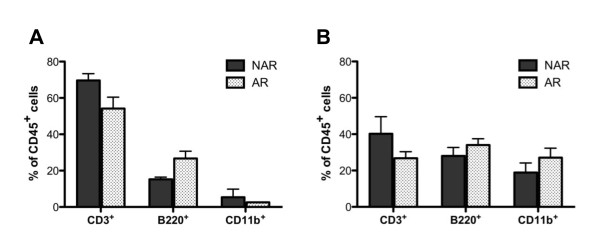
**Cellular composition of lymphoid organs in IIJ mice**. **(A) **and **(B) **Average number of cells of specific immune lineages in **(A) **lymph nodes and **(B) **spleens of AR or NAR IIJ mice based on flow cytometric analysis. Error bars represent SEM. Live cells were gated on and the amount of each cell type (CD3^+ ^= T cell, B220^+ ^= B cell and CD11b^+ ^= myeloid/granulocyte) was determined as the percentage of CD45^+ ^cells. All AR mice were more than seven weeks old and had been arthritic for a minimum of four weeks. NAR mice were littermates or age-matched within two weeks (*n *= 4 to 5 mice/group). Groups were compared by two-way analysis of variance, and cellular composition varied significantly in the lymph nodes (*P *< 0.05) but not in the spleens.

To determine whether any lymphocyte population was expanded and possibly activated in the AR mice, cells were isolated from the spleens and leg-draining lymph nodes of IIJ littermates and stained for flow cytometric analysis. Though the cellular makeup of the spleens did not differ significantly between the AR and NAR IIJ mice, a significant difference was found in the lymph nodes (*P *< 0.05) (Figure 6). The relative percentage of CD3^+ ^T cells was lower and the relative percentage of B220^+ ^B cells was higher in the AR mice compared to NAR littermates.

**Figure 7:**
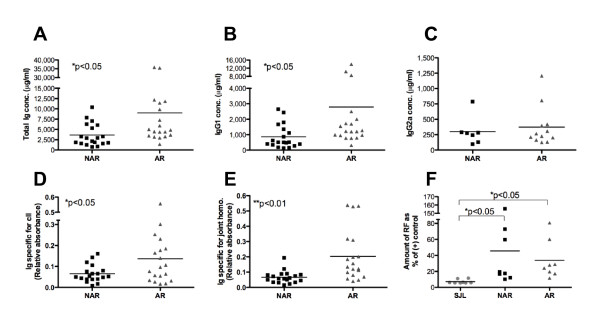
**Serum immunoglobulin levels in IIJ mice and age-matched SJL/J controls**. Serum was collected and Ig measured by ELISA. **(A) **through **(C) **Average levels of **(A) **total Ig, **(B) **IgG1, **(C) **IgG2b, **(D) **anti-collagen type II Ig and **(E) **anti-joint homogenate Ig. AR and NAR IIJ mice were littermates and were between 87 and 207 days old. No correlations between antibody level and age, sex, disease severity or days since disease onset were found (data not shown). **(F) **Amount of total Ig rheumatoid factor (RF) in NAR and AR littermates and age-matched SJL/J controls. All mice were 55 to 189 days old, and AR mice had had clinical disease for a minimum of four weeks. RF level did not correlate with total Ig or age (data not shown). Groups were compared by performing a *t*-test. Filled circles, squares, and triangles represent data points from NAR. Early AR, and Late AR mice, respectively.

Given this relative expansion in the B-cell population, serum Ig levels were measured by ELISA (Figure 7). Though the levels were variable, AR mice had, on average, higher levels of total Ig in their serum compared to NAR littermates (Figure 7A). Upon examination of individual isotypes, AR mice also had higher levels of IgG1 (Figure 7B) but similar levels of IgG2a (Figure 7C). To investigate antigen-specific reactivity, total Ig RF and Ig specific for collagen type II or joint homogenate were examined. Compared to NAR littermates, AR IIJ mice had, on average, higher serum levels of anti-collagen type II and anti-joint homogenate antibody (Figures 7D and 7E, respectively), though, again, significant variability was observed. AR and NAR mice had equivalent mean levels of RF, but levels in both groups were significantly higher than in age-matched SJL/J controls (Figure 7F). Ultimately, 7 (63.7%) of 11 NAR IIJ mice and 8 (72.7%) of 11 AR IIJ mice had RF levels at least twice the mean SJL/J RF level. Nevertheless, despite these differences in averages, there were AR mice without higher levels of total Ig, IgG1, RF or anti-collagen or anti-joint homogenate antibodies, calling into question a pathogenic role for antibodies in disease.

Therefore, sera from AR mice were adoptively transferred into SJL/J mice to determine whether serum alone could transfer disease. Three hundred microliters of filtered and pooled sera from either AR or NAR mice were injected intraperitoneally into SJL/J mice, which were then tracked for disease. The mice were followed for over two months, but no mice (zero of six with NAR serum injected into SJL/J mice and zero of seven with AR serum into SJL/J mice) developed arthritis.

### A large proportion of CD4^-^CD8^- ^(double-negative) T cells in the lymph nodes of arthritic IIJ mice

**Figure 8:**
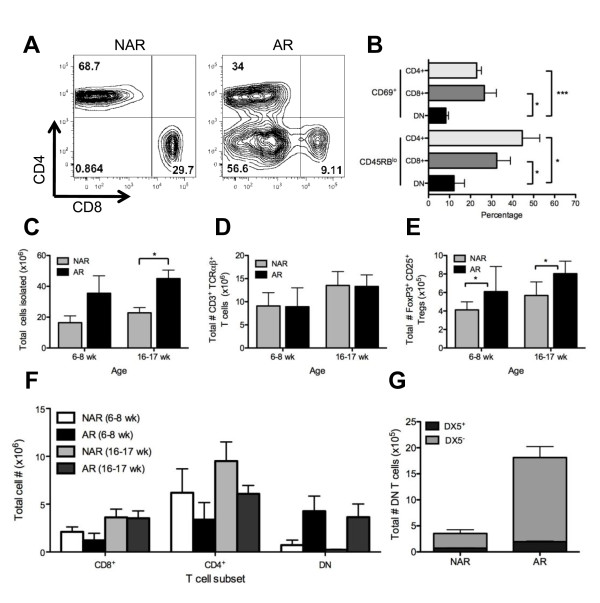
**Presence of double-negative (DN) T cells in lymph nodes of arthritic IIJ mice**. Cells were isolated from the leg-draining lymph nodes of AR and NAR littermates. They were stained and analyzed by flow cytometry. **(A) **Representative CD4 vs. CD8 density plots. Live CD3^+ ^TCRα/β^+ ^cells were gated on, and the percentage in each quadrant is shown. **(B) **Percentage of cells of each T-cell subset with activation/memory phenotype in arthritic mice (> 7 weeks old and arthritic for a minimum of 4 weeks) (*n *= 4 to 6 mice). **(C) **through **(F) **Comparison of absolute numbers of **(C) **cells isolated, **(D) **CD3^+ ^TCRαβ^+ ^T cells, **(E) **CD4^+ ^CD25^+ ^Foxp3^+ ^T-regulatory cells and **(F) **single positive (CD4^+ ^and CD8^+^) and DN (CD4^-^CD8^-^) T cells. For cell subsets, absolute numbers were back-calculated from percentages and number of total cells isolated. **(G) **Number of DN T cells that expressed the natural killer T-cell marker DX5 (*n *= 3 for each group in **(C) **through **(G)**). Data are means ± SEM. Groups were compared by performing a *t*-test (*P < 0.05, ***P < 0.001). Similar results were found in the spleens (data not shown).

Given the relative decrease in the proportion of T cells in the lymph nodes of the AR mice, T cells and subpopulations were examined in more detail (Figure 8). Cells from the leg-draining lymph nodes of young (6- to 8-week-old) and old (16- to 17-week-old) IIJ mice were isolated, counted, stained with antibody mixtures and analyzed by flow cytometry. Though the total number of cells isolated was elevated in AR mice compared to their NAR IIJ mice littermates (Figure 8C), the total number of α/β T cells was unchanged (Figure 8D), suggesting that the relative decrease in the percentage of CD3^+ ^cells (Figure 6) was more likely due to an expansion of B220^+ ^B cells than to a contraction of CD3^+ ^T cells specifically. γ/δ T cells were rare and were not different between groups (data not shown).

Nevertheless, α/β T cell subpopulations were significantly altered. The majority of the T cells in the lymph nodes of AR mice were, surprisingly, CD4^-^CD8^-^, or double-negative (DN) (Figure 8A), a population extremely rare in normal mice. While the number of DN T cells was expanded in young and old AR mice, the number of CD4^+ ^T cells was decreased, showing a loss of this subset specifically (Figure 8F). A similar, though less pronounced trend was also observed in the spleen (data not shown). To determine whether the decrease in CD4^+ ^T cells resulted from a loss of T-regulatory cells (Tregs), CD25^+^Foxp3^+ ^cells were examined (Figure 8E). AR mice in fact had more Tregs in their lymph nodes than their NAR littermates, suggesting an expansion of this population during chronic joint inflammation. This increase was seen in both 6- to 8-week-old and 16- to 17-week-old mice, and similar results were found when identifying Tregs on the basis of CD25 and GITR expression (data not shown). While the functionality of the Tregs is not known, the joint inflammation does not appear to be a result of a decline in this regulatory population.

The DN T cells were examined further for signs of activation and for additional lineage markers that might suggest their origins. To determine their activation and/or memory phenotype, CD69 upregulation (a reflection of recent activation) and CD45RB downregulation (a reflection of previous activation) were compared between T-cell subsets in AR mice (Figure 8B). While 25% to 45% of CD4^+ ^and CD8^+ ^T cells showed robust signs of activation, activation was low in the DN subset, calling into question whether the DN cells have a direct pathogenic role. Finally, since natural killer (NK) T cells lack CD4 and CD8 expression but are positive for the α/β TCR, the DN cells were examined for NK cell markers. More than 85% of the DN T cells lacked the pan-NK cell marker DX5 (Figure 8G), and therefore their origin is still under investigation.

### Typhlocolitis and other extraarticular manifestations of inflammation in IIJ mice

**Figure 9:**
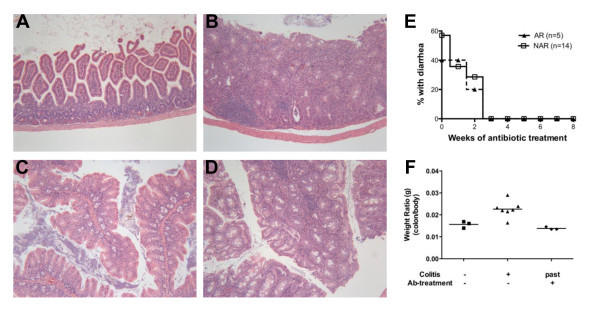
**Typhlocolitis in a subset of IIJ mice**. H & E-stained tissue sections of paraffin-embedded **(A) **and **(B) **colon and **(C) **and **(D) **cecum from IIJ mice (total magnification, ×100). **(A) **and **(C) **Noninflamed tissue from a NAR, 98-day-old IIJ mouse and **(B) **and **(D) **inflamed tissue from its AR littermate. Images are representative of sections from seven mice (see Table 3 for details). **(E) **Effective treatment of diarrhea with antibiotic-containing mouse chow. Mice were started on mouse chow and checked weekly for signs of diarrhea. **(F) **Colon:body weight ratios (g:g) for three groups of IIJ mice: mice that had never had diarrhea, mice with active diarrhea and mice that used to have diarrhea but had been successfully treated with antibiotics.

In the F5 generation, a subset of IIJ mice started developing diarrhea, which is possibly indicative of inflammatory bowel disease (IBD). Joint inflammation often accompanies IBD in humans, and therefore a link between the two diseases has been suggested [42,43]. Though some mice seemed to have diarrhea chronically, most only showed signs transiently. H & E staining of the GI tracts revealed evidence of typhlocolitis (inflammation of the cecum and colon) in all the AR mice examined, regardless of whether they showed signs of diarrhea at the time of organ harvest (Table 3 and Figures 9A, 9B, 9C, 9D). Gut pathology was characterized by significant lymphocytic, plasmacytic and neutrophilic infiltration into the submucosa, as well as marked mucosal hyperplasia and crypt abscess formation (Figures 9A, 9B, 9C, 9D). Though the two NAR IIJ mice examined showed no histological signs of typhlocolitis, more careful tracking of the occurrence of diarrhea through five generations of IIJ mice revealed that both AR and NAR IIJ mice developed diarrhea (Table 4). Of 273 mice tracked for IBD, 40% showed signs of diarrhea at least once at their weekly scoring for joint inflammation. Of the mice that had diarrhea, 66 (60%) of 110 also had arthritis. While this subset of IIJ mice showed the significant female bias in arthritis incidence (*P *< 0.05), no sex bias was observed in the incidence of diarrhea. SJL/J sentinel mice that received bedding from all the IIJ cages on the same rack never developed diarrhea or arthritis, indicating that IBD was specific to the IIJ strain.

**Table 3:** Extraarticular inflammation in arthritic IIJ mice

				Inflammation score^a^				
				
IIJ mouse	Arthritis	Diarrhea^b^	Age, days	Colon/cecum	Lung	Liver	Kidney	Heart
1	Y	Y	192	+++	-	-	-	-
2	Y	Y	98	+++	++	-	-	-
3	Y	N	147	+	+	+	+	-
4	Y	N	219	++	+	+	++	+
5	Y	Y	277	+++	-	++	+	-
6	N	N	98	-	-	-	-	-
7	N	N	219	-	-	-	-	-

^a^(-) no inflammation, (+) mild to minimal inflammation, (++) moderate inflammation, (+++) severe inflammation; ^b^at time of organ harvest.

**Table 4:** Incidence of inflammatory bowel disease in IIJ strain^a^

Disease manifestation	Total, *n *(%) (*n *= 273)	Females, *n *(%) (*n *= 131)	Males, *n *(%) (*n *= 142)
Arthritis	112 (41%)	64 (49%)	48 (34%)
Diarrhea	110 (40%)	49 (37%)	61 (43%)
Arthritis and diarrhea	66 (24%)	35 (27%)	31 (22%)

^a^Mice were considered to have inflammatory bowel disease if they ever showed signs of diarrhea at time of weekly scoring for joint inflammation.

To screen for possible GI pathogens, eight IIJ mice (five AR and three NAR) and four sentinel SJL/J mice from the colony were sent to the Comparative Pathology Laboratory at the University of California, Davis. All the IIJ mice, regardless of arthritis status, tested positive for a *Helicobacter *sp: six had *H. rodentium *and two had a *Helicobacter *sp. of undetermined type. They did not test positive for other GI tract pathogens, including pinworm. None of the SJL/J sentinels tested positive for *Helicobacter *spp. Believed to be a part of the normal intestinal flora, *H. rodentium *was originally identified in 1997, when it was isolated from the intestines of asymptomatic laboratory mice [44]. It was later found to cause mild to moderate colitis in IL-10-deficient mice [45] and severe IBD only when the mice were coinfected with other, more pathogenic *Helicobacter *spp. [45-47]. Treating IIJ mice with food tablets containing a four-antibiotic drug cocktail (0.06% amoxicillin, 0.0138% metronidazole, 0.0037% bismuth and 0.0004% omeprazole) resolved their diarrhea within three weeks (Figure 9E), and after eight weeks of treatment, the IIJ mice tested negative for all *Helicobacter *spp. (data not shown). While IIJ mice with active diarrhea had an elevated colon:body weight ratio, indicating inflammation, antibiotic treatment caused the ratio to normalize and become similar to the ratios of IIJ mice that had never had diarrhea (Figure 9F). While this finding does not prove that *Helicobacter *spp. infection specifically induced typhlocolitis in the IIJ mice, the correlation suggests that it might.

To determine whether the IIJ mice had evidence of inflammation in tissues other than the joints and the colon, various organs were harvested, stained with H & E and examined in a blinded fashion by a trained veterinary histopathologist. In addition to typhlocolitis, other extraarticular inflammation was observed in all of the AR mice, though the organs involved and the severity of inflammation were inconsistent (Table 3). Inflammation was noted in the lungs of three of five AR animals (two with mild to minimal inflammation and one with focal pleuritis). A similar number also showed inflammation in the liver and/or kidneys. Inflammation severity ranged from minimal to moderate, although one AR IIJ mouse had pronounced moderate interstitial nephritis and fibrosis in the kidneys. Finally, severe vasculitis was seen in the vertebrae of the oldest IIJ arthritis mouse (data not shown). The NAR IIJ mice showed no signs of inflammation in any organ (Table 3). Therefore, similarly to humans with RA, the AR IIJ mice had extraarticular manifestations of disease, indicating pathology of a systemic nature.

## Discussion

In this report, we describe a new strain of mouse, which we have named the IIJ strain, that spontaneously develops chronic inflammatory, and possibly autoimmune, arthritis that shares many similarities with human RA and other mouse models of arthritis. Though visible swelling was most evident in the large distal joints of IIJ mice, histological hallmarks of arthritis, including bone and cartilage erosion, invasive synovial hyperproliferation and immune cell infiltration were observed in all joints of the hindlimbs and forelimbs (Figure 1). Overall, the histopathology was similar to that described in previously published mouse models of autoimmune arthritis [32,34-36]. In addition, the predominantly neutrophilic and lymphocytic infiltration into the inflamed IIJ joints (Figure 4) parallels the high number of neutrophils and T cells present in the inflamed synovial fluid of RA patients [48,49]. Also similar to RA, inflammation was not restricted solely to the joints of IIJ mice, but was systemic in nature. Elevation of serum proinflammatory cytokines (Figure 5) and histological signs of inflammation, ranging from mild to severe, were observed in various organs (Table 3). Extraarticular manifestations of disease have also been noted in IL-1R antagonist-deficient [50] and SKG mice [36]. Finally, the IIJ mice also share serological similarities with RA and other mouse models. Compared to NAR littermates, AR IIJ mice had mildly elevated serum Ig, which is due, at least partially, to elevated IgG1, a classic Th2-induced isotype (Figure 7). RA patients also have slightly increased serum Ig levels [51], and researchers who have studied several spontaneous mouse models of arthritis, including the K/BxN [32], gp130^Y759F^[35] and IL-1R antagonist deficiency models [34], have reported increases in IgG1 specifically. Importantly, autoantibodies were also detected in our study, as many IIJ mice were positive for RF and antibodies specific to joint components (Figure 7).

The arthritis incidence in IIJ mice also displayed the sex bias common to many complex autoimmune diseases, such as RA, multiple sclerosis and systemic lupus erythematosus (SLE) [52]. RA incidence was higher in females than in males (38% vs. 28%; *P *< 0.001) (Table 1), though ultimately clinical severity and mean day of onset did not differ significantly. The sex bias appeared to be specific for the arthritis phenotype, since the incidence of typhlocolitis was similar between male and female IIJ mice (Table 4). As most models reach 100% incidence in both sexes, no other spontaneous mouse model of arthritis has found such a sex bias, although researchers who have studied both the SKG [36] and gp130^Y759F ^models [35] have noted more severe arthritis in females. A female bias in incidence was observed in collagen-induced arthritis in humanized HLA-DR4-transgenic mice [53] and was attributed to both hyperactive B cells and HLA-DR4-restricted antigen presentation in female mice and increased numbers of Tregs and B-regulatory cells in male mice [54]. The reason for the sex bias in arthritis incidence in IIJ mice remains unknown.

It remains unclear whether arthritis in the IIJ mice is truly autoimmune. The similarities to RA and other mouse models of autoimmune arthritis outlined above would strongly argue in favor of this scenario. The relative expansion of the B-cell population, elevated serum Ig and presence of autoantibodies are also strongly suggestive that this is true. Nevertheless, all AR IIJ mice did not have levels of serum autoantibodies that exceeded those of NAR littermates (Figure 7), and the increase might simply be a side effect of chronic inflammation. Some murine models of arthritis have shown elevated levels of Ig and autoantibodies, but the arthritis was lymphocyte-independent. For example, joint inflammation still occurs in the *N*-ethyl-*N-*nitrosourea-induced mutants *Ali5 *and *Ali18*, even when crossed to Rag1^-/- ^backgrounds [55,56]. Finally, transfer of sera from AR IIJ mice failed to induce disease in SJL/J hosts, suggesting that antibodies alone are not sufficient to cause disease. It is possible that not enough serum was transferred and/or that it was not transferred by the appropriate method (for example, intravenous rather than intraperitoneal injection). Nevertheless, the same transfer protocol successfully transfers disease in antibody-dependent arthritis models such as the K/BxN model [57].

The appearance of a high percentage of DN T cells in the peripheral lymphoid organs does indicate that the T-cell compartment in the AR IIJ mice is significantly disrupted (Figure 8). DN T cells are characteristic of SLE in humans as well as in murine models of SLE, such as the MRL/lpr mouse, a strain that also gets arthritis [58,59]. Since the peripheral DN T cells in MRL/lpr mice are B220^+ ^[59] and those in IIJ mice are B220^- ^(data not shown), it is unlikely that these mouse populations are identical. The origin of the DN T cells in IIJ mice remains unclear. It does not appear to caused by an expansion of NK T cells, as DN T cells lack the pan-NK cell marker DX5 (Figure 8G), and their high expression of CD3 and the TCR suggests that they are mature. The fact that CD4^+ ^T cells decrease in number as DN T cells increase does suggest that the DN T cells might be CD4^+ ^cells that have downregulated CD4. While downregulation of TCR coreceptors by viruses have been well documented [60], it has also been proposed as a natural mechanism of peripheral tolerance [61,62]. Autoreactive T-cell clones can downregulate CD4 upon long-term stimulation *in vitro *[63], and self-specific TCR-transgenic mice can downregulate coreceptors to control autoimmunity [64]. Nevertheless, the fact that the IIJ DN T cells largely lack an activated and/or memory phenotype (Figure 8B) argues against coreceptor downregulation due to chronic activation. The existence of a DN T-cell-regulatory population to control self-reactivity in humans and mice has been proposed [65,66], and the expanded DN T-cell population in mice bearing the homozygous Fas^lpr ^or FasL^gld ^mutations has been found to have regulatory properties [67]. The ontogeny and functional ability of the IIJ DN T cells are currently under investigation.

The gene mutations and background alleles that might cause or modify the arthritis phenotype in the IIJ strain have yet to be identified. It is unlikely that the phenotype is related to either the 5B6 TCR transgenes or the Thy1.1 allele that were present in the line from which the IIJ strain was derived. A low percentage of peripheral blood T cells in the IIJ mice expressed Vβ6, the variable region used in the 5B6-transgenic TCRβ chain, and none of the IIJ mice (> 1,000 animals) have shown clinical or histological signs of EAE (for example, loss of muscle tonicity, hindlimb paralysis or inflammation in the central nervous system). Also, the arthritis occurs regardless of Thy1 status, as we have identified AR and NAR IIJ mice that are Thy1.1^+^Thy1.2^-^, Thy1.1^-^Thy1.2^+ ^and Thy1.1^+^Thy1.2^+ ^(data not shown). Both SJL/J and FVB background alleles likely contribute to arthritis development. The strain from which the IIJ mice were derived was predominantly SJL/J in background. Statistically, however, with a minimum of only six generations of backcrossing to SJL/J after generating the 5B6-transgenic mice on the FVB background, a small percentage of FVB alleles must be present in the IIJ strain. The fact that multiple attempts to backcross the AR IIJ mice further to SJL/J only decreased arthritis incidence also argues in favor of FVB-influencing alleles. Since backcrossing AR IIJ mice to FVB inbred mice also resulted in low AR incidence, SJL/J alleles must also be important.

The pedigree does indicate that the arthritis phenotype is a complex, non-Mendelian trait and, together with the female bias in incidence, is clearly sex-influenced. The incidence of 32% in the F1 generation after crossing the "founder" AR male with the SJL/J females argues for a dominant phenotype (Figure 2). Nevertheless, the fact that crossing two NAR IIJ mice yielded a similar incidence of arthritis suggests that the trait is also incompletely penetrant, since the NAR parents must harbor the disease-promoting alleles but do not display the trait. While it is also possible that a combination of multiple alleles must be inherited to develop the disease, the relative consistency of incidence regardless of the phenotype of the parents argues against simple segregation of multiple alleles. Furthermore, at the time of the writing of this article, the IIJ mice had been intercrossed for 14 generations and the overall incidence had not increased, despite the fact that most loci should be fixed after that much inbreeding. Therefore, while genes are undoubtedly influencing the phenotype, other factors, such as microbial stimulation of the innate immune system, or even stochastic events, such as the formation of the T- and B-cell repertoires through random rearrangements in the TCR and Ig genes, might also be involved.

Some bacteria, such as *Borrelia burgdorferi *and *Staphylococcus aureus*, or microbial products such as zymosan, a crude fungal extract of β-glycans, can induce arthritis in susceptible mouse strains [25-27]. While it cannot be completely ruled out, there is little evidence that the arthritis in the IIJ mice is caused purely by an infectious agent. The mice are maintained in a barrier facility, and arthritis incidence does not cluster by cage. Also, SJL/J mice housed in the same cage as AR IIJ mice never develop arthritis, and SJL/J sentinel mice that receive portions of bedding from over 20 cages of IIJ mice housed on the same rack also never develop arthritis. The IIJ breeding colony was transported between institutions (from Northwestern University to Westmont College) in the F3 and F4 generation, but arthritis incidence did not significantly change after the move. No viral, bacterial or parasitic pathogen has been consistently found in the IIJ strain, aside from the mice with *H. rodentium *infection, and the AR IIJ mice that have been treated with antibiotics to clear the *Helicobacter *sp. have remained AR. Nevertheless, even spontaneous arthritis can be highly influenced by microbial flora. For example, the SKG strain has a high incidence of arthritis in conventional caging conditions and a very low incidence of arthritis in SPF conditions, unless they are injected with zymosan [68]. It is not known whether a similar innate immune system would trigger an increased incidence of arthritis in the IIJ mice.

While *H. rodentium *does not appear to cause the arthritis phenotype, it seems likely that it is responsible for or at least contributes to typhlocolitis, since *H. rodentium *infection is prevalent in both AR and NAR IIJ mice and treatment with antibiotics cleared the *Helicobacter *sp. and resolved the diarrhea and gut inflammation (Figure 9). As mentioned previously, *H. rodentium *was identified only 13 years ago, when it was isolated from asymptomatic laboratory mice [44]. Few studies have since investigated its pathogenicity. In experimental monoinfections, *H. rodentium *did not cause cecal lesions or significantly change gut gene expression in either immunocompetent A/JCr mice or SCID (severe combined immunodeficiency) mice, while, in parallel experiments, a related species (*H. hepaticus*) did [46]. Several studies have found that *H. rodentium *exacerbates gastrointestinal inflammation when coinfected with other *Helicobacter *spp. [45-47], although no other *Helicobacter *spp. were found in the IIJ mice. If this bacterial species alone is responsible for the typhlocolitis, it might reflect a particular predisposition to proinflammatory immune responses in IIJ mice. RA as well as IBDs such as ulcerative colitis and Crohn's disease are chronic inflammatory diseases, and genome-wide association studies have identified genetic loci that increase the risk for both [69-72]. Although several case studies have been published about RA patients who developed IBD [73,74], IBD is not considered a typical extraarticular manifestation of RA [2,3]. In contrast, joint inflammation occurs in 20% of patients diagnosed with IBD, though it is classified as peripheral or axial spondyloarthritis [42,43] and is typically seronegative for RF and anticyclic citrullinated peptide antibodies [75,76].

## Conclusion

Overall, the data presented identify the IIJ strain as a new murine model of inflammatory, possibly autoimmune, arthritis. The IIJ strain is similar, both histologically and serologically, to RA and other murine models of autoimmune arthritis. In addition, the increased incidence of arthritis in female IIJ mice makes it a potentially important model for the study of the underlying causes of the sex bias in autoimmunity. Nevertheless, notable differences do exist between the disease in IIJ mice and human RA. For example, joint swelling in RA is typically symmetric in humans [1,77], while it is asymmetric in many of the IIJ mice (Figure 3). Also, the accumulation of DN T cells in the periphery, which is so marked in IIJ mice (Figure 8), is a hallmark of SLE, not RA [58]. Despite these differences, the IIJ strain is a new experimental tool with which to investigate the genetic and immunological mechanisms of arthritis.

## Abbreviations

AR: arthritic; CBA: cytometric bead array; DMEM: Dulbecco's modified Eagle's medium; FCS: fetal calf serum; H & E: hemotoxylin and eosin; HBSS: Hanks' balanced salt solution; Ig: immunoglobulin; IIJ: inherited inflamed joints; IL: interleukin; NAR: nonarthritic; PBS: phosphate-buffered saline; RA: rheumatoid arthritis; RF: rheumatoid factor; TCR: T-cell receptor; TNF-α: tumor necrosis factor α.

## Competing interests

The authors declare that they have no competing interests.

## Authors' contributions

IA conducted the cytokine CBA assays and the majority of the flow cytometry. JW was critical in compiling and analyzing the clinical data, performed most of the Ig ELISAs and designed and conducted preliminary transfer experiments. CK designed and conducted the outcrossing experiments. CR and ET designed and carried out the studies related to typhlocolitis. KS was invaluable in completing the transfer experiments and the flow cytometry experiments. CS researched and completed the RF ELISAs. SM helped conceive of the study and participated in experimental design, interpretation of data and revision of the manuscript. EM also helped conceive of the study, performed all preliminary experiments, participated in experimental design and data interpretation, coordinated the study, trained all student researchers and wrote the manuscript.
